# A Room‐Temperature Verwey‐type Transition in Iron Oxide, Fe_5_O_6_

**DOI:** 10.1002/anie.201914988

**Published:** 2020-01-30

**Authors:** Sergey V. Ovsyannikov, Maxim Bykov, Sergey A. Medvedev, Pavel G. Naumov, Anton Jesche, Alexander A. Tsirlin, Elena Bykova, Irina Chuvashova, Alexander E. Karkin, Vadim Dyadkin, Dmitry Chernyshov, Leonid S. Dubrovinsky

**Affiliations:** ^1^ Bayerisches Geoinstitut Universität Bayreuth Universitätsstrasse 30 95447 Bayreuth Germany; ^2^ Max Planck Institute for Chemical Physics of Solids 01187 Dresden Germany; ^3^ FSRC “Crystallography and Photonics” RAS Leninskiy Prospekt 59 Moscow 119333 Russia; ^4^ Experimental Physics VI Center for Electronic Correlations and Magnetism Institute of Physics University of Augsburg 86135 Augsburg Germany; ^5^ Deutsches Elektronen-Synchrotron (DESY) 22603 Hamburg Germany; ^6^ M. N. Miheev Institute of Metal Physics of Ural Branch of Russian Academy of Sciences 18 S. Kovalevskaya Str. Yekaterinburg 620137 Russia; ^7^ Institute for Solid State Chemistry of Ural Branch of Russian Academy of Sciences 91 Pervomayskaya Str. 620990 Yekaterinburg Russia; ^8^ Swiss-Norwegian Beamlines at the European Synchrotron Radiation Facility 38000 Grenoble France; ^9^ Geophysical Laboratory, Carnegie Institution of Washington 5251 Broad Branch Rd. NW 20015 Washington, DC USA

**Keywords:** charge ordering, high pressure, iron oxides, transition metal oxide, Verwey transition

## Abstract

Functional oxides whose physicochemical properties may be reversibly changed at standard conditions are potential candidates for the use in next‐generation nanoelectronic devices. To date, vanadium dioxide (VO_2_) is the only known simple transition‐metal oxide that demonstrates a near‐room‐temperature metal–insulator transition that may be used in such appliances. In this work, we synthesized and investigated the crystals of a novel mixed‐valent iron oxide with an unconventional Fe_5_O_6_ stoichiometry. Near 275 K, Fe_5_O_6_ undergoes a Verwey‐type charge‐ordering transition that is concurrent with a dimerization in the iron chains and a following formation of new Fe−Fe chemical bonds. This unique feature highlights Fe_5_O_6_ as a promising candidate for the use in innovative applications. We established that the minimal Fe−Fe distance in the octahedral chains is a key parameter that determines the type and temperature of charge ordering. This model provides new insights into charge‐ordering phenomena in transition‐metal oxides in general.

## Graphical abstract

**Charge and order**: Crystals of novel mixed‐valent iron oxide with an unconventional Fe_5_O_6_ stoichiometry were synthesized and investigated. Near 275 K, Fe_5_O_6_ demonstrates a Verwey‐type charge‐ordering transition that is concurrent with a pronounced dimerization and the formation of new Fe−Fe chemical bonds. This unique feature highlights Fe_5_O_6_ as a highly promising candidate for use in innovative applications.

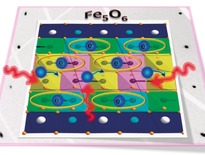

Strongly correlated oxides, with their unusual optoelectronic and magnetic properties related to interacting electrical charges, are of considerable interest from both fundamental and applied perspectives.^1^, ^2^, ^3^, ^4^, ^5^, ^6^, ^7^, ^8^ The existence of unique phase transitions or crossover points in these materials at temperatures close to room temperature would open a way to their use in various innovative applications,^1^, ^2^, ^3^, ^4^, ^5^, ^6^, ^7^ where properties of materials can be switched by external stimuli like heat, stress (strain), the electric field, and others. However, such cases are rather rare, particularly among simple oxides of inexpensive transition metals that are commercially most attractive. Presently, vanadium dioxide (VO_2_), demonstrating an abrupt metal–insulator transition whose temperature point can be moderately tuned about 340 K,^1^, ^2^, ^3^ is considered as a basic candidate for the use in prospective appliances using controlled switching of its electrical properties. A number of novel devices utilizing the metal–insulator transition in VO_2_ has been already reported, including field‐effect transistors,^4^ various ultrafast optoelectronic switches,^5^, ^6^ and memory elements.^7^ Recently, a photoreversible metal–semiconductor phase transition was discovered at room temperature in the *λ*‐phase of Ti_3_O_5_,^8^ thereby highlighting this material as an alternative candidate for optoelectronic switches. Magnetite (Fe_3_O_4_), one of the most common iron compounds, also shows an abrupt change in its electrical properties at the charge‐ordering Verwey transition,^9^, ^10^ but the transition temperature of 120 K is restrictively low for use in oxide electronics.^11^, ^12^ Transition‐metal oxides that could reversibly and significantly change their physical properties at temperatures close to room temperature would be of great interest, but none of the binary oxide phases reported so far demonstrate that.^13^ Recent studies using high‐pressure/high‐temperature (HP‐HT) synthesis techniques reported on the discovery of novel binary iron oxides with unconventional stoichiometries (for example, Fe_4_O_5_, Fe_5_O_6_, Fe_5_O_7_, Fe_7_O_9_, Fe_9_O_11_),^14^, ^15^, ^16^, ^17^, ^18^ which are structurally related to the family of calcium ferrites, $Ca{Fe}_{n}^{2+}{Fe}_{2}^{3+}O_{4+n}$ ,^19^ where the Ca sites are filled with Fe^2+^ ions. These novel mixed‐valence iron oxides are of fundamental importance, but their physicochemical properties and technological potential have yet to be investigated.

Here, we synthesize crystals of a novel iron oxide with the unconventional Fe_5_O_6_ stoichiometry,^15^ and explore their structural and physical properties. We establish that at a temperature of 275 K, Fe_5_O_6_ undergoes an unusual phase transition, with charge ordering realized through the formation of novel Fe−Fe chemical bonds between two adjacent ions in the linear octahedral chains. This leads to a pronounced dimerization within these chains and to significant changes in the electrical resistivity and other physical properties. Even though the phase transition in Fe_5_O_6_ is not as abrupt as the metal–insulator transition in VO_2_,^1^, ^2^, ^3^, ^4^ it demonstrates novel features that can stimulate the development of atomic‐scale switches. For example, by using appropriate optical or other techniques, one can manipulate individual Fe−Fe dimers in thin single‐crystalline films of Fe_5_O_6_ whose orientation coincides with the dimerization direction. From the more fundamental perspective, we establish that the minimal Fe−Fe distance in the octahedral chains is the key parameter that determines the type and temperature of the charge‐ordering transition. This model provides new insights into charge‐ordering phenomena in both iron oxides and transition‐metal oxides in general.

We synthesized the Fe_5_O_6_ samples under moderate HP‐HT conditions using a multi‐anvil press at Bayerisches Geoinstitut (BGI; see experimental procedures in the Supporting Information)^20^, ^21^ and selected a set of high‐quality single crystals for the present investigation (Figure 1 a), including a temperature‐dependent single‐crystal X‐ray diffraction at the European synchrotron radiation facility.^22^ The Fe_5_O_6_ samples adopted the orthorhombic *Cmcm* structure (No. 63), where the iron cations fill the linear chains of both the octahedra (crystallographic sites Fe1 and Fe2) and the trigonal prisms (Fe3; Supporting Information, Figure S1 and Table S1).^15^ A bond‐valence‐sum (BVS)^23^ analysis (see experimental procedures in the Supporting Information) of the Fe−O bond lengths in this structure returned BVS values of 2.57(6), 2.43(7), and 1.97(4) for Fe1, Fe2, and Fe3, respectively. Thus, the more spacious prismatic Fe3 sites are exclusively filled with Fe^2+^, likewise, the two inequivalent octahedral sites are filled with mixed Fe^2+^/Fe^3+^ ions.

**Figure 1:**
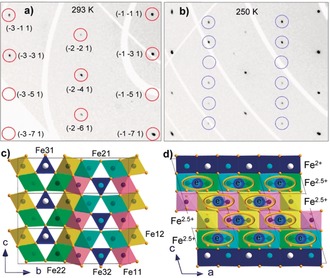
a), b) Examples of reciprocal lattice planes (*hk*1) of Fe_5_O_6_ demonstrating the appearance of superlattice reflections (highlighted by blue circles) upon the phase transition from the original Fe_5_O_6_‐I phase [at 293 K, (a)] to the low‐temperature Fe_5_O_6_‐II phase [at 250 K, (b)]. Positions of the basic structural reflections in a) are highlighted by red circles and indexed. c), d) Two projections of the crystal structure of the Fe_5_O_6_‐II phase showing the formation of the Fe−Fe dimers with one shared electron in the chains of the octahedrally coordinated iron ions. The cations labels are given in (c).

Upon cooling below room temperature, a well‐ordered array of superlattice reflections appeared in the diffraction patterns of the Fe_5_O_6_ single crystals (Figure 1 a,b). This indicates the emergence of an additional structural order. We solved the crystal structure of this low‐temperature phase, labelled as Fe_5_O_6_‐II, in the *P*2_1_/*m* monoclinic space group (No. 11; Table S2). The Fe−Fe distances in the octahedral chains in this phase displayed a pronounced separation into pairs, resulting in the formation of dimers (Figure 1 d). In Figure 2 a, one can see that upon the phase transition, a single Fe−Fe periodicity of 2.877 Å along the octahedral iron chains turns into a periodicity of two alternating distances; these distances correspond to the dimers (short distance) and the gaps between them (long distance). A BVS analysis of the Fe−O bond lengths in this lattice indicates that the iron ions filling the octahedral sites keep their non‐integer valences of about +2.5 (Figure 2 b). Hence, we can conclude that the dimers in the Fe_5_O_6_‐II structure are composed mainly of a pair of Fe^2+^ and Fe^3+^ in which one electron simultaneously belongs to both iron ions (Figure 1 d). Thus, the phase transition leads to the stabilization of a formal fractional oxidation state of ≈+2.5 for the octahedrally coordinated iron cations. This fractional oxidation state is not a result of mathematical averaging of Fe^2+^ and Fe^3+^ valences, because such a model does not explain the formation of the dimers.

**Figure 2:**
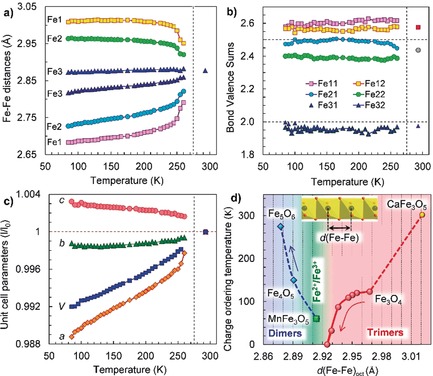
Temperature dependencies of a) two characteristic Fe−Fe distances within each chain of the iron cations along the *a*‐axis (Figure 1 d), b) bond‐valence sums of the iron cations, and c) the unit‐cell parameters. Two Fe−Fe distances in the octahedral chains Fe1 and Fe2 in (a) correspond to the dimers (short distance) and gaps between them (long distance). The dashed vertical line at 275 K indicates the midpoint of the transition. d) Dependence of the charge‐ordering transition temperature (*T*_CO_) for Fe_5_O_6_ (this work), Fe_4_O_5_,^28^ MnFe_3_O_5_,^31^ Fe_3_O_4_,^10^, ^34^ and CaFe_3_O_5_ 29, 30 on the minimal Fe−Fe distances in their octahedral iron chains. The arrows indicate the pressure‐induced changes found for Fe_3_O_4_ 34 and Fe_4_O_5_.^32^

The normal Fe−Fe bond length value in α‐Fe metal is 2.48 Å, whereas in complexes containing metal clusters it was always found to be longer, in the range of 2.5–2.7 Å and above, signifying a half‐bond resonance.^24^, ^25^ The shortest Fe−Fe distances of ≈2.78 Å in the Fe_5_O_6_‐II structure at 260 K were found in the Fe1 chains of Fe11 and Fe12 octahedra (Figure 1 c,d); with decreasing temperature, these distances decrease to ≈2.68 Å (Figure 2 a). This behavior of the crystal structure of the Fe_5_O_6_‐II phase points to the formation of the Fe−Fe bonds and their strengthening upon cooling below the transition point.

We measured temperature dependencies of the electrical resistivity for two crystals of Fe_5_O_6_ and observed an abrupt increase in the resistivity below 280–290 K (Figure 3 a). A derivative of one of these curves suggested a midpoint of this transition at 275 K (inset in Figure 3 a). Thus, the structural distortion in Fe_5_O_6_ is accompanied by a significant increase in the electrical resistivity. At room temperature, the electrical resistivity of these Fe_5_O_6_ crystals amounted to ≈7 mΩ cm (Figure 3 a,c). This value is almost the same as the one found earlier in high‐quality single crystals of magnetite (≈4 mΩ cm).^26^ Hence, Fe_5_O_6_ is a good electrical conductor at room temperature, and as magnetite, it can be characterized by a high concentration of low‐mobile carriers associated with hopping charges.^26^ The Fe_5_O_6_‐II phase is apparently semiconducting with an activation energy of about 0.1 eV (inset in Figure 3 a), which corresponds to a band‐gap value of *E*_g_ *=*2 *E*_a_≈0.2 eV. This is double the value of the charge‐ordered phase of magnetite, where *E*_g_≈0.1 eV.^27^

**Figure 3:**
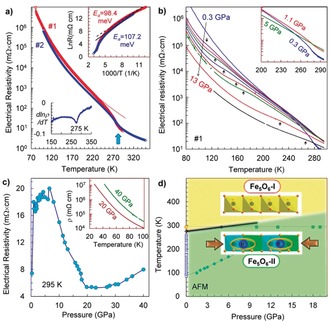
Temperature dependencies of the electrical resistivity of Fe_5_O_6_ a) for two samples (#1, #2) at 0.3 GPa and b) for sample #1 at different pressures. The insets in a) show the determination of the transition midpoint (275 K) and the activation energy in the charge‐ordered phase. The inset in (b) demonstrates that an applied pressure shifts the transition point above room temperature. c) Pressure dependence of the electrical resistivity at 295 K. Two temperature curves of the electrical resistivity at 20 and 40 GPa are given in the inset in (c). d) Pressure–temperature phase diagram based on structural and electrical resistivity data. The two colored regions correspond to the different phases. The line starting from 100 K corresponds to the kink in the electrical resistivity curves in (b), and potentially, it may be linked to the antiferromagnetic (AFM) transition.

With application of pressure, the charge‐ordering transition seen in the electrical resistivity curve of Fe_5_O_6_ shifts above room temperature (inset in Figure 3 b). In line with this finding, direct X‐ray diffraction studies of an Fe_5_O_6_ single crystal, compressed at room temperature above 10 GPa, confirmed that the sample adopts the charge‐ordered Fe_5_O_6_‐II structure (Figure 3 d). A positive pressure coefficient of this charge‐ordering transition temperature, *T*_CO_, together with an apparent possibility to control the *T*_CO_ magnitude by a minor uniaxial contraction or stretching along the dimerization direction (Figure 3 d), indicate that Fe_5_O_6_ could be utilized in innovative near‐room‐temperature applications.

The phase transition in Fe_5_O_6_ detected by X‐ray diffraction and electrical‐transport measurements also manifests itself in the temperature dependence of the magnetic susceptibility, *χ*(*T*), which reveals a shallow but well‐defined minimum around *T*≈270 K (Figure 4 a). A strong increase in these *χ*(*T*) curves observed upon cooling below 270 K suggests the formation of local magnetic moments following charge localization on the iron dimers. Non‐linear inverse susceptibility indicates deviations from the Curie–Weiss behavior in this temperature range (insets in Figure 4 b,c). Nevertheless, from the average slope one can tentatively estimate an effective magnetic moment of *μ_eff_*=1.6 μ_B_ per formula unit in both samples. A sharp drop in the *χ*(*T*) curves marks a purely antiferromagnetic transition with the Néel temperature of *T*_N_=100 K in the applied field of *μ*_0_ *H*=1 T (Figure 4 b,c). We also observed a pronounced field dependence of the *χ*(*T*) curves below *T*_N_ in higher fields. The isothermal magnetization measurements showed a series of metamagnetic transitions around *T*_N_ for 5 T<*μ*_0_ *H*<7 T (Figure 4 d). This remarkable sensitivity of Fe_5_O_6_ to the applied field can be caused by electron‐sharing between the two Fe atoms, which would change the nature of the magnetic orbital and the interaction of magnetic electrons with the field.

**Figure 4:**
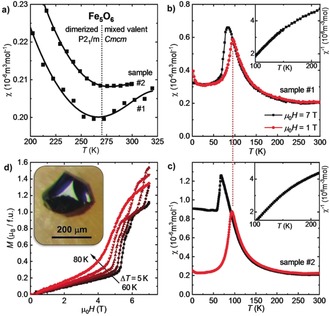
a) Temperature dependencies of the magnetic susceptibility of Fe_5_O_6_, *χ*(*T*)=*M*/*H*, near the structural phase transition in a magnetic field of *μ*_0_ *H*=7 T. b), c) A sharp drop in the *χ*(*T*) curves indicates antiferromagnetic ordering at *T*_N_≈100 K in a field of 1 T. The Fe_5_O_6_‐II phase demonstrates local‐moment behavior, albeit with a minor deviation from the Curie–Weiss law [insets in (b,c)]. A second magnetic transition may take place around 60 K, where the susceptibility becomes temperature‐independent. A field dependence of *χ*(*T*) below *T*_N_ is reflected in a series of *meta*‐magnetic transitions observed in the isothermal magnetization measurements in (d). The inset in (d) shows one of the single crystals of Fe_5_O_6_ that was used for these magnetic measurements.

A variety of the charge‐ordering features observed in octahedral networks of iron oxides studied to date^10^, ^28^, ^29^, ^30^, ^31^, ^32^ suggests the existence of several competing mechanisms of these phenomena. In general, one expects that the type of charge ordering is mainly influenced by the electron count and spin arrangement, should the magnetic ordering precede the charge‐ordering transition. In Figure 2 d, we plot the charge‐ordering temperatures (*T*_CO_) of several iron oxides vs. the shortest Fe−Fe distances, *d*(Fe−Fe)_oct_, in the edge‐shared linear octahedral chains. This plot indicates a crossover near *d*(Fe−Fe)_oct_≈2.91 Å separating the regions of short and long Fe−Fe distances, optimal for the formation of dimers and trimers, respectively (Figure 2 d). Interestingly, three oxides—Fe_4_O_5_,^28^ CaFe_3_O_5_,^29^, ^30^ and MnFe_3_O_5_,^31^ crystallizing in similar structures comprising the divalent ions (Fe^2+^, Mn^2+^, Ca^2+^) in the prisms and nearly identically charged octahedra (Fe^2.67+^ on average)—demonstrate different types of charge order. Upon the charge ordering in the least dense CaFe_3_O_5_ with *d*(Fe−Fe)_oct_=3.021 Å or 3.014 Å, the iron atoms shift by only ≈0.01 Å and form loosely packed iron trimers,^29^, ^30^ suggesting that the formation of these trimers is rather magnetically mediated.^33^ MnFe_3_O_5_ with *d*(Fe−Fe)_oct_=2.914 Å lies near the crossover point and exhibits a conventional Fe^2+^/Fe^3+^ charge separation.^31^ A spectacular case was revealed in Fe_4_O_5_, which showed a competition between the trimeric and dimeric types of charge ordering, leading to a complex incommensurately modulated structure of the charge‐ordered phase.^28^ This charge order in Fe_4_O_5_ could be realized due to a compromise between the high Fe^2.67+^ average charge in the octahedra, which is optimal for the formation of trimers (Fe^3+^−Fe^2+^−Fe^3+^),^10^ and a too short *d*(Fe−Fe)_oct_=2.891 Å distance that is more suitable for the formation of dimers (Figure 2 d). Remarkably, an applied pressure was found to suppress this charge‐ordered phase of Fe_4_O_5_, to squeeze out the excess charge from the octahedra to the prisms, and to stabilize a purely dimeric order with the strongly enhanced *T*_CO_.^32^ Thus, Fe_5_O_6_ with its shorter *d*(Fe−Fe)_oct_=2.877 Å distance, optimally charged octahedral ions (Fe^2.5+^ on average), and with the high *T*_CO_=275 K temperature further enhanced under pressure fits well to the trend found for Fe_4_O_5_ (Figure 2 d). In contrast, it was found on the example of magnetite that the trimeric order below 120 K is suppressed by an applied hydrostatic pressure of 8 GPa,^34^ although it can persist to higher pressures upon quasi‐hydrostatic compression.^35^

The coupling of the charge ordering and dimerization we observed in Fe_5_O_6_ suggests that electron–phonon interactions play a key role in this phase transition, as proposed earlier for the Verwey transition in magnetite.^36^ Our results facilitate an understanding of the underlying mechanisms of this phenomenon in different iron oxides, and indicate new perspectives for a large group of transition‐metal oxides and other materials.^37^, ^38^, ^39^ Phase transitions associated with dimerization (for example, Peierls transitions) can find various practical applications, but their utilization is usually hindered by low transition temperatures.^39^ Thus, Fe_5_O_6_ and its solid solutions, (Fe,*M*)Fe_4_O_6_,^40^ in which the charge‐ordering transition temperatures may be tuned by certain dopants that fill the prismatic sites and affect the Fe−Fe distances in the octahedral chains (Figure 1 d), are potential materials for various innovative applications, for example, stress‐controlled elements,^41^ switches, or memory devices.

## Conflict of interest

The authors declare no conflict of interest.

## Supporting information

As a service to our authors and readers, this journal provides supporting information supplied by the authors. Such materials are peer reviewed and may be re‐organized for online delivery, but are not copy‐edited or typeset. Technical support issues arising from supporting information (other than missing files) should be addressed to the authors.

Supplementary
