# Supplementary material for: A Room‐Temperature Verwey‐type Transition in Iron Oxide, Fe5O6
Source: Angew Chem Int Ed Engl. 2020 Jan 30;59(14):5632–6. doi: 10.1002/anie.201914988 (PMC7154779; doi:10.1002/anie.201914988)
Supplement: Supplementary file 1 — Supplementary [file ANIE-59-5632-s001.pdf]

Angewandte Chemie
Eine Zeitschrift der Gesellschaft Deutscher Chemiker

Supporting Information

A Room-Temperature Verwey-type Transition in Iron Oxide, Fe_5O_6

Sergey V. Ovsyannikov, Maxim Bykov, Sergey A. Medvedev, Pavel G. Naumov, Anton Jesche, Alexander A. Tsirlin, Elena Bykova, Irina Chuvashova, Alexander E. Karkin, Vadim Dyadkin, Dmitry Chernyshov, and Leonid S. Dubrovinsky*

anie_201914988_sm_miscellaneous_information.pdf

Table of Contents

Experimental Procedures......S2
Results......S2
References......S6
Author Contributions......S6

Experimental Procedures

Preparation and characterization of Fe_5O_6

Single crystals of Fe_5O_6 were synthesized at high-pressure high-temperature (HP-HT) conditions at 14-16 GPa and 1200-1400 °C in 1200-tonne Multi-Anvil Presses at BGI.[1] For their preparation we used stoichiometric mixtures of Fe_3O_4 (Aldrich, 99.99% purity) and Fe (99.99%). The samples were synthesized during several hours. We utilized Re sample capsules, $LaCrO_3$ heaters, W3Re/W25Re thermocouples, and octahedral containers; other details were similar to those described in previous works.[1,2] Notice here, that the phase stability regions of Fe_5O_6 and of another oxide, Fe_4O_5 are essentially overlapped,[3-5] and for this reason, the synthesis products were usually mixtures of both Fe_5O_6 and Fe_4O_5 crystals. Using single crystal X-ray diffraction, we selected good crystals of Fe_5O_6 for the present study. Chemical purity of these crystals was verified by scanning electron microscopy (SEM) with a LEO-1530 instrument.

Single crystal X-ray diffraction of Fe_5O_6

For selection of the Fe_5O_6 crystals, we used a high-brilliance Rigaku diffractometer (Mo Kα radiation) equipped with Osmic focusing X-ray optics and Bruker Apex CCD detector. The temperature-dependent single crystal X-ray diffraction studies from 293 K down to 80 K were carried out at SNBL (The Swiss-Norwegian Beam Line, ESRF, Grenoble, France) with a wavelength of 0.6884 Å. We collected the intensity data using a single-axis diffractometer equipped with a Pilatus 2M pixel detector by 360° φ scans ($\Delta\varphi$ = 1°).[6] For processing of these data (which included determination of orientation matrix and indexing, peak intensities integration, background evaluation, and absorption correction), we employed the *CrysAlis*^{Pro} 171.37.35 program. For solution of crystals structures, we used *SHELXT* software.[7] The structure refinements were performed within the *OLEX*^{2} software using *SHELXL*.[7,8] “CSD 1979456-1979460 contains the supplementary crystallographic data for this paper. These data can be obtained free of charge from the Cambridge Crystallographic Data Centre via www.ccdc.cam.ac.uk/structures. We analysed the Fe-O bond lengths in the both crystal structures of Fe_5O_6 using the well-known bond valence sum (BVS) method,[9] in which a bond valence is determined as $S_{ij} = \exp[(R_{ij}-d_{ij})/b_0]$, where d_{ij} is the distance between atoms i and j, R_{ij} is the bond valence parameter (empirically determined distance for this cation-anion pair), and b_0 is an empirical parameter of about 0.37 Å. Then, a bond valence sum is determined as a sum of these individual bond valences, $V_i = \Sigma(S_{ij})$.[9] In these calculations, we used b_0=0.37 Å and the bond-valence parameters R_{ij} determined at ambient conditions for Fe^{2+}–O and Fe^{3+}–O bonds as 1.734 Å and 1.759 Å, respectively.[9]

Measurements of magnetic properties of Fe_5O_6

For measurements of magnetic properties, we employed a Quantum Design MPMS3. The measurements were performed on two crystals with well-developed facets that were mounted in arbitrary orientation. We determined the sample masses by averaging of results of 25 measurements, performed on a balance with a nominal accuracy of 1 μg. We obtained values of m=9 μg and m=11 μg for sample #1 and #2, respectively. Alternative estimation of the sample masses based on their volume values yielded m~9 μg. A tiny amount of some ferromagnetic impurity inclusion was subtracted by assuming a linear field dependence of magnetization at room temperature. In both samples a contribution to the total moment amounted to 1.1×10^{-9} Am^2, which could be explained by a potential presence of impurity grains like Fe_3O_4 of roughly 0.1% of the total sample mass.

Measurements of electrical resistivity of Fe_5O_6

We measured electrical resistivity of Fe_5O_6 in a temperature range between 1.7 K and 300K using a direct current van der Pauw technique in a diamond anvil cell, from ambient pressure and up to 40 GPa. Diameters of flat working surfaces of the diamond anvils and of a hole in a gasket were of 500 μm and 200 μm, respectively. A tungsten gasket was insulated with a cubic BN/epoxy mixture. For electrodes we utilized a platinum (Pt) foil with a thickness of 5 μm. For these measurements, we selected two flat high-quality single crystals of Fe_5O_6 with linear sizes of about 70 μm and loaded them in a sample chamber filled with NaCl pressure-transmitting medium. Pressure values were determined using the ruby scale for small chips of ruby placed near with the sample. Upon initial compression, in both samples we observed the smooth jumps in the electrical resistivity curves below 280 K by about one order of magnitude (Figure 3a).

Results

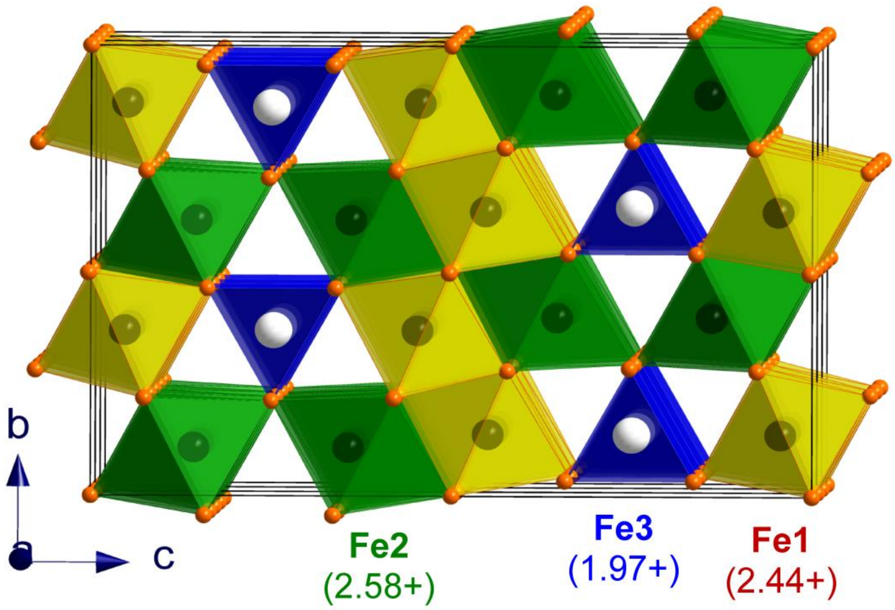

Figure S1. Crystal structure of Fe_5O_6 at ambient conditions. The labels of iron ions and their bond valence sums (BVS)[9] values are given near the ions.

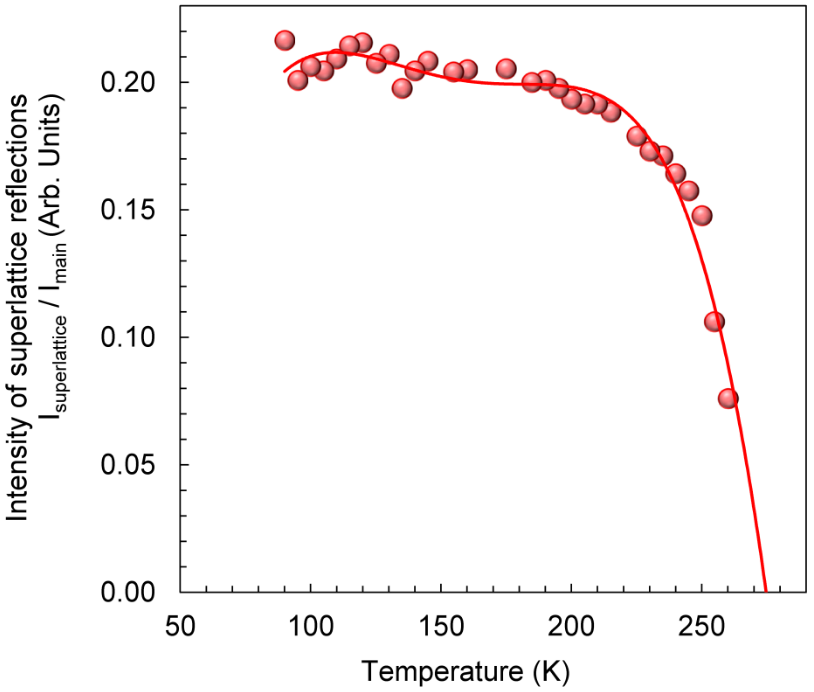

Figure S2. Temperature dependence of the superlattice reflections intensity in Fe_5O_6. The data are normalized by the main reflections intensity.

Table S1. Crystal structure parameters of Fe_5O_6 at ambient conditions (Fe_5O_6–I phase)

**Crystal data**			
Chemical formula	Fe_5O_6		
M_r	375.25		
Crystal system, space group	Orthorhombic, *Cmcm*		
Temperature (K)	293		
a, *b*, *c* (Å)	2.8774 (2)	9.9173 (7)	15.3402 (15)
V (Å^3)	437.75 (6)		
Z	4		
Radiation type	Synchrotron, λ = 0.69489 Å		
□ (mm^{-1})	15.21		
Crystal size (mm)	0.1 × 0.05 × 0.05		

**Data collection**	
Diffractometer	Pilatus @ SNBL
Absorption correction	Multi-scan
T_{min}, T_{max}	0.241, 1.000
No. of measured, independent and observed [*I* > 2□(*I*)] reflections	1394, 438, 433
R_{int}	0.019
(sin □/□)$_{max}$ (Å^{-1})	0.764

**Refinement**	
$R[F^2 > 2□(F^2)]$, $wR(F^2)$, S	0.038, 0.103, 1.14
No. of reflections	438
No. of parameters	37
□ρ_{max}, □ρ_{min} (e Å^{-3})	0.97, -0.84

**Atomic coordinates**	x	y	z
Fe1	0	0.36754(4)	0.04721(2)
Fe2	0	0.10765(5)	0.14280(3)
Fe3	0	0.13702(7)	0.75
O1	0	0.2786(2)	0.57689(15)
O2	0	0.4610(2)	0.16076(14)
O3	0	0.2107(3)	0.25
O4	0	0	0

Table S2. Crystal structure parameters of Fe_5O_6 at low-temperature conditions (Fe_5O_6–II phase).

Temperature (K)		85	135	215	245
**Crystal data**					
Crystal system, space group		$P2_1/m$	$P2_1/m$	$P2_1/m$	$P2_1/m$
Lattice parameters (Å)	*a:*	5.6902 (2)	5.70520 (13)	5.72228 (11)	5.73104 (11)
	b:	15.3214 (6)	15.3169 (4)	15.3226 (5)	15.3258 (3)
	c:	5.17979 (19)	5.17697 (12)	5.1747 (3)	5.17315 (10)
β (°)		105.934 (4)	105.957 (2)	106.002 (2)	106.057 (5)
V (Å^3)		434.23 (3)	434.96 (2)	436.14 (3)	436.65 (2)
μ (mm^{-1})		15.33	15.31	15.27	15.25
Crystal size (mm)		0.1 × 0.05 × 0.05	0.1 × 0.05 × 0.05	0.1 × 0.05 × 0.05	0.1 × 0.05 × 0.05
**Data collection**					
Absorption correction		Multi-scan	Multi-scan	Multi-scan	Multi-scan
T_{min}, T_{max}		0.025, 0.053	0.434, 1.000	0.258, 1.000	0.570, 1.000
No. of measured, independent and observed [$I > 2\sigma(I)$] reflections		2018, 2018, 1817	2031, 2031, 1806	2020, 2020, 1766	2024, 2024, 1742
$(\sin \theta/\lambda)_{max}$ (Å^{-1})		0.767	0.767	0.767	0.766
**Refinement**					
$R[F^2 > 2\sigma(F^2)]$, $wR(F^2)$, S		0.067, 0.217, 1.10	0.045, 0.150, 1.09	0.056, 0.188, 1.14	0.046, 0.156, 1.12
$\Delta\rho_{max}$, $\Delta\rho_{min}$ (e Å^{-3})		2.24, −2.48	1.97, −2.12	1.81, −2.45	2.65, −1.80
**Atomic coordinates**					
Fe11	x/a:	0.05096(12)	0.05069(9)	0.05216(10)	0.05345(8)
	y/b:	0.04825(3)	0.04818(2)	0.04804(3)	0.04798(2)
	z/c:	0.76053(9)	0.76056(7)	0.76122(8)	0.76166(7)
Fe12	x/a:	0.41822(12)	0.41907(9)	0.42046(10)	0.42203(8)
	y/b:	0.54695(3)	0.54689(2)	0.54679(3)	0.54686(2)
	z/c:	0.23055(10)	0.23038(7)	0.23063(9)	0.23116(7)
Fe21	x/a:	0.20953(13)	0.20710(10)	0.20548(11)	0.20399(9)
	y/b:	0.14207(4)	0.14213(3)	0.14237(3)	0.14242(3)
	z/c:	0.29029(12)	0.29010(9)	0.28950(11)	0.28911(9)
Fe22	x/a:	0.31312(12)	0.31388(10)	0.31232(11)	0.31144(9)
	y/b:	0.64245(4)	0.64256(3)	0.64269(3)	0.64267(3)
	z/c:	0.71728(12)	0.71715(9)	0.71725(11)	0.71701(9))
Fe31	x/a:	0.17635(16)	0.17953(14)	0.18004(16)	0.18067(12)
	y/b:	0.75	0.75	0.75	0.75
	z/c:	0.22086(18)	0.21994(13)	0.22098(16)	0.22147(14)
Fe32	x/a:	0.31891(15)	0.31572(14)	0.31603(16)	0.31606(12)
	y/b:	0.25	0.25	0.25	0.25
	z/c:	0.77951(18)	0.77752(13)	0.77630(16)	0.77614(14)
O11	x/a:	0.3866(4)	0.3867(4)	0.3867(4)	0.3871(3)
	y/b:	0.07545(19)	0.07571(14)	0.07499(14)	0.07576(13)
	z/c:	0.0537(6)	0.0540(5)	0.0544(5)	0.0538(5)
O12	x/a:	0.1032(5)	0.1022(4)	0.1024(4)	0.1029(3)
	y/b:	0.57685(18)	0.57672(13)	0.57581(14)	0.57640(12)
	z/c:	0.9333(6)	0.9332(4))	0.9347(5)	0.9361(5)

O21	x/a:	0.0143(5)	0.0192(4)	0.0195(5)	0.0202(4)
	y/b:	0.1610(2)	0.16080(14)	0.16022(15)	0.16087(13)
	z/c:	0.5764(7)	0.5775(5)	0.5762(6)	0.5766(5)
O22	x/a:	0.4807(5)	0.4785(5)	0.4792(5)	0.4789(4)
	y/b:	0.6608(2)	0.66046(14)	0.65981(14)	0.66037(13)
	z/c:	0.4147(7)	0.4142(5))	0.4171(6)	0.4179(5)
O31	x/a:	0.1402(9)	0.1441(7)	0.1427(8)	0.1432(6)
	y/b:	0.25	0.25	0.25	0.25
	z/c:	0.0876(9)	0.0880(7)	0.0881(9)	0.0848(7)
O32	x/a:	0.3613(8)	0.3574(7)	0.3593(8)	0.3582(6)
	y/b:	0.75	0.75	0.75	0.75
	z/c:	0.9197(9)	0.9193(7)	0.9193(9)	0.9217(8)
O4	x/a:	0.2535(3)	0.2527(3)	0.2518(3)	0.25143(19)
	y/b:	0.00270(9)	0.00233(8)	0.00215(8)	0.00184(6)
	z/c:	0.5068(3)	0.5067(2)	0.5060(2)	0.50566(17)

References

[1] D. J. Frost, B. T. Poe, R. G. Trønnes, C. Liebske, A. Duba, D. C. Rubie, *Phys. Earth Planet. Inter.* **2004**, *143–144*, 507–514.

[2] S. V. Ovsyannikov, M. Bykov, E. Bykova, D. P. Kozlenko, A. A. Tsirlin, A. E. Karkin, V. V. Shchennikov, S. E. Kichanov, H. Gou, A. M. Abakumov, R. Egoavil, J. Verbeeck, C. McCammon, V. Dyadkin, D. Chernyshov, S. van Smaalen, L. S. Dubrovinsky, *Nature Chem.* **2016**, *8*, 501-508

[3] B. Lavina, P. Dera, E. Kim, Y. Meng, R. T. Downs, P. F. Weckf, S. R. Sutton, Y. Zhao, *Proc Nat. Acad. Sci. USA* **2011**, *108*, 17281–17285.

[4] B. Lavina, Y. Meng, *Science Advances* **2015**, *1*, e1400260.

[5] R. Myhill, D. O. Ojwang, L. Ziberna, D. J. Frost, T. Boffa Ballaran, N. Miyajima, *Contrib. Mineral. Petrol.* **2016**, *171*, 51.

[6] V. Dyadkin, Ph. Pattison, V. Dmitriev, D. Chernyshov, *J. Synchrotron Rad.* **2016**, *23*, 825–829.

[7] G. M. Sheldrick, *Acta Crystallogr. C* **2015**, *71*, 3–8.

[8] O. V. Dolomanov, L. J. Bourhis, R. J. Gildea, J. A. K. Howard, H. Puschmann, *J. Appl. Crystallogr.* **2009**, *42*, 339–341.

[9] N. E. Brese, M. O'Keffee, *Acta Cryst*. *B* **1991**, *47*, 192–197.

Author Contributions

S.V.O. and L.S.D. initiated and designed the study. S.V.O. synthesized the Fe_5O_6 samples. M.B., E.B., I.C., V.D., and D.C. performed the single crystal X-ray diffraction study. M.B. analyzed the diffraction data. S.A.M., P.G.N., and A.E.K. measured the electrical properties. A.J. and A.A.T. measured the magnetic properties. S.V.O. wrote the paper with contributions from all co-authors.
